# Biologic Cost per Effectively Treated Rheumatoid Arthritis Patient in a Large Managed Care Population: A Retrospective Cohort Study

**DOI:** 10.36469/9830

**Published:** 2015-09-17

**Authors:** Tao Gu, Neel Shah, Gaurav Deshpande, Derek H. Tang, Debra F. Eisenberg, David J. Harrison

**Affiliations:** 1 HealthCore, Wilmington, DE; 2 Amgen Inc., Thousand Oaks, CA

**Keywords:** managed care, cost, effectiveness, biologics, rheumatoid arthritis

## Abstract

**Background:** Until recently, the lack of clinical outcomes information for rheumatoid arthritis (RA) in administrative claims databases limited their use in comparative effectiveness research. A validated claims-based algorithm has been developed to estimate the effectiveness of biologics for RA, allowing for estimation of cost and effectiveness in the same database.

**Objectives:** To implement a validated claims-based effectiveness algorithm in a US managed care claims database to compute the 1-year biologic cost per effectively treated patient among first-line biologics approved for moderate-to-severe RA (abatacept, adalimumab, certolizumab pegol, etanercept, golimumab, and infliximab).

**Methods:** This retrospective cohort study used administrative claims data for individuals in the HealthCore Integrated Research Database (HIRDSM). The first claim for a first-line biologic between July 1, 2009, and January 31, 2013, after 6 months of continuous enrollment, was defined as the index event and date. Patients were aged 18-63 years on the index date and had at least one claim for RA in the 6-month pre- index period. Biologic costs included plan and patient paid amounts on claims for the biologic drug and administration. The algorithm defined effectiveness during the 12-month post-index period as achieving all six of the following: high adherence (medication possession ratio ≥80% or infusions consistent with the product label); no increase in biologic dose or decrease in dosing interval; no new biologic; no new nonbiologic disease-modifying antirheumatic drug; no new or increased oral glucocorticoid use; and ≤1 glucocorticoid injection. Cost per effectively treated patient was calculated as the total biologic cost (drug and administration) divided by the number of patients categorized by the algorithm as effectively treated.

**Results:** The cohort comprised 4844 patients (mean age 48.6 years, 76.4% female). Average first-year biologic cost ranged from $14 795 (golimumab) to $19 520 (abatacept). Average first-year biologic cost per effectively treated patient was significantly lower for etanercept ($50 217) than for golimumab ($56 427, p<0.001) adalimumab ($56 879, p<0.001), abatacept ($68 062, p<0.001), certolizumab pegol ($76 427, p<0.001), and infliximab ($95 126, p<0.001).

**Conclusions:** In this application of a validated claims-based algorithm to a large managed care population, etanercept had the lowest 1-year biologic cost per effectively treated patient among first-line biologics.

## BACKGROUND

In the United States, the lifetime risk of developing rheumatoid arthritis (RA) is 3.6% for women and 1.7% for men.[1] RA, a long-term condition characterized by inflammation of the joints and surrounding tissues, can also affect other organs.[2,3] It carries with it a risk of complications including, but not limited to, rheumatoid lung, hardening of the arteries (rheumatoid vasculitis), spinal injury, and swelling and inflammation of the outer lining of the heart (pericarditis) and of the heart muscle (myocarditis).[4] Additionally, patients with RA have increased likelihood of developing cancer, infection, cataracts, osteoporosis, or psoriasis.[5] Although the cause of RA is unknown, infections, genes, hormonal changes, and smoking are risk factors that have been linked to the disease.[3]

RA typically requires lifelong treatment, including pharmacotherapy, physical therapy, exercise, and possibly surgery. Pharmacotherapy is often used to reduce inflammation in the joints to relieve pain and prevent or slow joint damage in patients with RA. Available pharmacotherapy includes corticosteroids, oral disease-modifying antirheumatic drugs (DMARDs), and biologics.[6,7] Biologics play an important role in the treatment of RA by helping to regulate the body's inflammatory process and inhibiting damage. Biologics approved for the treatment of RA in the United States include tumor necrosis factor (TNF) inhibitors (anti-TNFs) such as adalimumab, certolizumab pegol, etanercept, golimumab, and infliximab; and agents with other mechanisms of action such as abatacept, anakinra, rituximab, and tocilizumab. Biologics differ in their method of administration, dosing level, dosing schedule, and whether they are approved for first- line use (without requiring DMARD failure as a prerequisite) or second-line use (requiring at least one DMARD failure). Adalimumab, anakinra, certolizumab pegol, etanercept, and golimumab are administered by subcutaneous injection, whereas infliximab and rituximab are administered by intravenous infusion. Abatacept, golimumab, and tocilizumab are currently available in both subcutaneous and intravenous dosage forms.

Until recently, the lack of clinical outcomes information for RA in administrative claims databases limited their use in comparative effectiveness research. A claims-based algorithm was developed to estimate the effectiveness of biologics for RA; the algorithm was validated using Veterans Administration data[8] and was further evaluated with commercial claims data.[9] The objective of this study was to implement the validated claims-based algorithm in a United States managed care database to compute the 1-year biologic cost per effectively treated patient among first-line biologics approved for moderate-to-severe RA (abatacept, adalimumab, certolizumab pegol, etanercept, golimumab, and infliximab).

## METHODS

### Data Source

This retrospective cohort study used administrative data contained in the HealthCore Integrated Research Database (HIRD[SM]). The HIRD contains medical and pharmacy claims data from the largest commercially insured population in the United States. The HIRD includes a broad and geographically diverse spectrum of longitudinal claims data, including 14 commercial health plans distributed across the southeastern, mid-Atlantic, central, and western regions of the United States. Data contained within HIRD cover approximately 50 million patient lives. Fully adjudicated paid claims data are updated on a monthly basis, and nearly 100% of adjudicated medical claims become available within 3 months of the date when the service was rendered. The data for this analysis were accessed and analyzed in a manner that complied with The Health Insurance Portability and Accountability Act of 1996 (HIPAA) regulations, including those related to the privacy and security of individually identifiable health information.

## Patient Eligibility

The cohort included patients with at least one claim for abatacept, adalimumab, certolizumab pegol, etanercept, golimumab, or infliximab between July 1, 2009, and January 31, 2013. The index biologic was the first biologic that a patient used in this period following 180 days of continuous enrollment in a health plan covered in HIRD. The index date was the date on which the index biologic was initiated. The pre-index period for each patient included the 6 months prior to and including the index date. The post-index period for each patient included the 12 months after the index date.

To be included in the analysis, patients needed to be continuously enrolled in HIRD for 6 months before their index date, and their index date needed to occur after the Food and Drug Administration approval of the biologic for the treatment of RA. Patients were excluded from the analysis for any of the following reasons: age <18 years or >64 years; continuous enrollment for <12 months (<365 days) after the index date; claims for more than one biologic of interest on the index date; no RA diagnosis in the pre-index period; a diagnosis for another indication for one of these biologics (plaque psoriasis, psoriatic arthritis, ankylosing spondylitis, juvenile idiopathic arthritis, Crohn's disease, ulcerative colitis, non-Hodgkin's lymphoma, or chronic lymphocytic leukemia) in the pre-index period; a pharmacy claim with a National Drug Code or an intravenous biologic; a medical claim for a Healthcare Common Procedure Coding System for a subcutaneous biologic; use of any biologic during the pre-index period; or outlier dosing (more than twice the maximum allowed dose in the product labeling). Anakinra, rituximab, and tocilizumab were not included in the analysis because they were not approved for first-line use in RA.

## Effectiveness

The validated claims-based algorithm[8] was used to categorize the index biologic as effective if the patient met all six of the criteria throughout the 12-month post-index period. The index biologic was categorized as not effective if the patient failed any of the six criteria.

1. High adherence to index drug:
   a. Self-administered subcutaneous biologics: ≥80% adherence to therapy with adalimumab, certolizumab pegol, etanercept, or golimumab, calculated as a medication possession ratio (MPR). The MPR was defined as the sum of the days' supply of the medication, divided by the number of days between the first fill and the last refill, plus the days' supply of the last refill. A maximum of 14 days overlap was allowed. If the days' supply extended beyond the 12-month post-index period, it was capped at index date + 365 days.
   b. Intravenously infused biologics: Dosing consistent with the US prescribing information (≥14 abatacept infusions; ≥7 infliximab infusions).
2. No biologic switch or addition (ie, no claim for a non-index biologic) during the post-index period.
3. No new nonbiologic DMARD (methotrexate, sulfasalazine, leflunomide, or hydroxychloroquine) during the post-index period that the patient was not already taking during the pre-index period.
4. No increase in the biologic dose or decrease in the dosing interval:
   a. Adalimumab could not be increased to 40 mg once weekly.
   b. Etanercept could not be increased to 50 mg twice weekly.

c. For abatacept and infliximab, the difference between the ending and starting doses, with each dose rounded up to the nearest 100 mg, could not be ≥100 mg. The number of infliximab infusions could not exceed 120% of the number expected assuming a 0-, 2-, or 6-week load and an 8-week infusion schedule.

5. No more than one glucocorticoid joint injection (Current Procedural Terminology codes: 20600, 20605, 20610) on more than one unique calendar day between the index date + 90 days and the end of the post-index period.

6. No new or increased dose of an oral glucocorticoid:

a. Patients who received no prescriptions for oral glucocorticoids during the pre-index period could not receive oral glucocorticoids for >30 days between the index date + 90 days and the end of the post-index period.

b. Patients who received at least one prescription for an oral glucocorticoid in the pre-index period could not receive a cumulative glucocorticoid dose during the last 6 months of the post-index period that was >120% of the cumulative glucocorticoid dose during the pre-index period.

Pearson's correlation coefficients were computed to estimate the correlations between each criterion in the algorithm. Correlation coefficients between ±0.8 and ±1.0 translate to a very strong relationship; ±0.6 to ±0.8 translate to a strong relationship; ±0.4 to ±0.6 translate to a moderate relationship; ±0.2 to ±0.4 translate to a weak relationship; and ±0.0 to ±0.2 translate to a very weak or no relationship.[10]

### Cost Variables

Biologic costs were obtained from plan and patient paid amounts on claims for the biologic drug and administration during the 12-month post-index period for each patient who received that index biologic. Average biologic cost per effectively treated patient was calculated for each index biologic as the total biologic cost, divided by the number of patients categorized by the algorithm as effectively treated, as follows:

$$\text{Cost per Effectively Treated Patient} = \frac{\text{Average Cost per Treated Patient}}{\text{\% Effectively Treated Patients}}$$

$$= \frac{\text{Total Cost}}{\text{Number of Effectively Treated Patients}}$$

Average cost per effectively treated patient was compared between index biologics by using Student's *t*-test, after performing Bootstrap method, with etanercept as the comparator.

## RESULTS

### Study Population

A total of 4844 patients were included in the analysis. The main reasons for exclusion from the study population (Table 1) were no RA diagnosis in the 6 month pre-index period, use of any biologic in the pre-index period, and continuous enrollment for <12 months after the index date.

**Table 1.** Selection of Study Sample

| Reason for Attrition | Excluded n | Remaining n (%) |
|---|---|---|
| ≥1 biologic claim between July 1, 2009, and January 31, 2013 | | 66 876 |
| Not continuously enrolled 180 days pre-index | 4242 | 62 634 (93.7) |
| Index biologic claim date before FDA approval date | 0 | 62 634 (93.7) |
| Age <18 years | 1870 | 60 764 (90.9) |
| Not continuously enrolled 365 days after index | 15 281 | 45 483 (68.0) |
| >1 biologic on index date | 4 | 45 479 (68.0) |
| No RA diagnosis in pre-index period | 26 786 | 18 693 (28.0) |
| Plaque psoriasis diagnosis in pre-index period | 768 | 17 925 (26.8) |
| Psoriatic arthritis diagnosis in pre-index period | 808 | 17 117 (25.6) |
| Ankylosing spondylitis diagnosis in pre-index period | 468 | 16 649 (24.9) |
| JIA diagnosis in pre-index period | 337 | 16 312 (24.4) |
| NHL or CLL diagnosis in pre-index period | 153 | 16 159 (24.2) |
| Ulcerative colitis diagnosis in pre-index period | 174 | 15 985 (23.9) |
| Crohn's disease diagnosis in pre-index period | 244 | 15 741 (23.5) |
| Age >64 years | 2517 | 13 224 (19.8) |
| Appropriate source of claims* | 222 | 13 002 (19.4) |
| Biologic use in pre-index period | 7655 | 5347 (8.0) |
| Outlier dosing | 40 | 5307 (7.9) |
| Index biologic was rituximab or tocilizumab† | 463 | 4844 (7.2) |

CLL: chronic lymphocytic leukemia; FDA: Food and Drug Administration; JIA: juvenile idiopathic arthritis; NHL: non- Hodgkin's lymphoma; pre-index period: from 180 days before index date until index date; RA: rheumatoid arthritis

*Appropriate sources of claims were National Drug Codes for subcutaneous biologics and Healthcare Common Procedure Coding System for intravenous biologics

†Excluded because they were not approved for first-line use at the time of the analysis

Baseline characteristics of study patients are summarized by index biologic in Table 2. The mean age was 48.6 years (standard deviation, 10.2), and 76.4% of patients were female. Most of the patients were enrolled in a preferred provider organization (67.5%) or a health maintenance organization (27.0%). The most commonly used index biologic was etanercept (43.9%), followed by adalimumab (27.1%), infliximab (10.2%), abatacept (5.0%), golimumab (3.1%), and certolizumab pegol (2.1%). Baseline characteristics appeared to be similar across the index biologics.

### Biologic Cost and Effectiveness

Biologic costs and effectiveness per the validated claims-based algorithm are provided in Table 3. The average biologic cost in the first year, including the drug cost plus the administration cost, ranged from $14 795 for golimumab to $19 520 for abatacept. The percentage of effectively treated patients who achieved all six of the algorithm criteria throughout the first year of biologic treatment ranged from 19.3% for infliximab to 33.3% for etanercept. The average first-year cost per effectively treated patient was lowest for etanercept ($50 217), followed by golimumab ($56 427; $p<0.001$ vs etanercept by Student's t-test), adalimumab ($56 879; $p<0.001$), abatacept ($68 062; $p<0.001$), certolizumab pegol ($76 427; $p<0.001$), and infliximab ($95 126; $p<0.001$).

**Table 2.** Characteristics of the Study Population

| | Total (N = 4844) | Abatacept (n = 265) | Adalimumab (n = 1437) | Certolizumab Pegol (n = 111) | Etanercept (n = 2328) | Golimumab (n = 164) | Infliximab (n = 539) |
|---|---|---|---|---|---|---|---|
| Age, mean (SD) | 48.6 (10.2) | 50.5 (9.6) | 48.6 (10.1) | 49.0 (9.4) | 48.1 (10.4) | 49.2 (10.6) | 49.6 (9.6) |
| **Sex, n (%)** | | | | | | | |
| Female | 3699 (76.4) | 219 (82.6) | 1102 (76.7) | 93 (83.8) | 1737 (74.6) | 136 (82.9) | 412 (76.4) |
| Male | 1145 (23.6) | 46 (17.4) | 335 (23.3) | 18 (16.2) | 591 (25.4) | 28 (17.1) | 127 (23.6) |
| **Region, n (%)** | | | | | | | |
| Northeast | 681 (14.1) | 42 (15.8) | 206 (14.3) | 22 (19.8) | 324 (13.9) | 19 (11.6) | 68 (12.6) |
| Midwest | 1502 (31.0) | 82 (30.9) | 444 (30.9) | 22 (19.8) | 728 (31.3) | 39 (23.8) | 187 (34.7) |
| South | 1465 (30.2) | 77 (29.1) | 443 (30.8) | 34 (30.6) | 704 (30.2) | 52 (31.7) | 155 (28.8) |
| West | 1196 (24.7) | 64 (24.2) | 344 (23.9) | 33 (29.7) | 572 (24.6) | 54 (32.9) | 129 (23.9) |
| **Health plan type, n (%)** | | | | | | | |
| HMO | 1308 (27.0) | 57 (21.5) | 406 (28.3) | 33 (29.7) | 626 (26.9) | 31 (18.9) | 155 (28.8) |
| PPO | 3270 (67.5) | 187 (70.6) | 955 (66.5) | 73 (65.8) | 1581 (67.9) | 126 (76.8) | 348 (64.6) |
| Other | 266 (5.5) | 21 (7.9) | 76 (5.3) | 5 (4.5) | 121 (5.2) | 7 (4.3) | 36 (6.7) |
| **Physician specialty, n (%)** | | | | | | | |
| Rheumatology | 3754 (77.5) | 127 (47.9) | 1171 (81.5) | 87 (78.4) | 1913 (82.2) | 150 (91.5) | 306 (56.8) |
| Internal medicine | 101 (2.1) | 23 (8.7) | 13 (0.9) | 0 (0.0) | 28 (1.2) | 1 (0.6) | 36 (6.7) |
| Family physician | 43 (0.9) | 0 (0.0) | 14 (1.0) | 0 (0.0) | 24 (1.0) | 0 (0.0) | 5 (0.9) |
| Dermatology | 2 (0.0) | 1 (0.4) | 1 (0.1) | 0 (0.0) | 0 (0.0) | 0 (0.0) | 0 (0.0) |
| Other | 210 (4.3) | 46 (17.4) | 29 (2.0) | 1 (0.9) | 56 (2.4) | 3 (1.8) | 75 (13.9) |
| Unknown | 734 (15.2) | 68 (25.7) | 209 (14.5) | 23 (20.7) | 307 (13.2) | 10 (6.1) | 117 (21.7) |

HMO: health maintenance organization; PPO: preferred provider organization; SD: standard deviation

**Table 3.** Biologic Cost and Effectiveness per the Algorithm in Patients With Rheumatoid Arthritis

| | Abatacept (n = 265) | Adalimumab (n = 1437) | Certolizumab Pegol (n = 111) | Etanercept (n = 2328) | Golimumab (n = 164) | Infliximab (n = 539) |
|---|---|---|---|---|---|---|
| Index biologic cost | $19 520 | $17 456 | $16 525 | $16 696 | $14 795 | $18 354 |
| Effectively treated, n (%) | 76 (28.7) | 441 (30.7) | 24 (21.6) | 774 (33.3) | 43 (26.2) | 104 (19.3) |
| **Biologic cost per effectively treated patient** | | | | | | |
| Cost | $68 062 | $56 879 | $76 427 | $50 217 | $56 427 | $95 126 |
| p-value* | <0.001 | <0.001 | <0.001 | Reference | <0.001 | <0.001 |

*p-value versus etanercept by Student's t-test

The percentages of patients who achieved each of the algorithm criteria are shown in Figure 1. Patients were more likely to fail the criteria for high adherence and no dose increase than the other algorithm criteria. High adherence ranged from 27.9% for certolizumab pegol to 54.0% for infliximab and no dose increase ranged from 58.3% for infliximab to 100% for etanercept. Statistical comparisons were not conducted at the individual criterion level.

As shown in Table 4, the relationship between adherence and biologic switch showed a weak correlation of 0.3 and all other relationships between two individual criteria showed very weak correlations of 0.2 or less.

**Figure 1.** Percentage of Patients Meeting Individual Algorithm Criteria

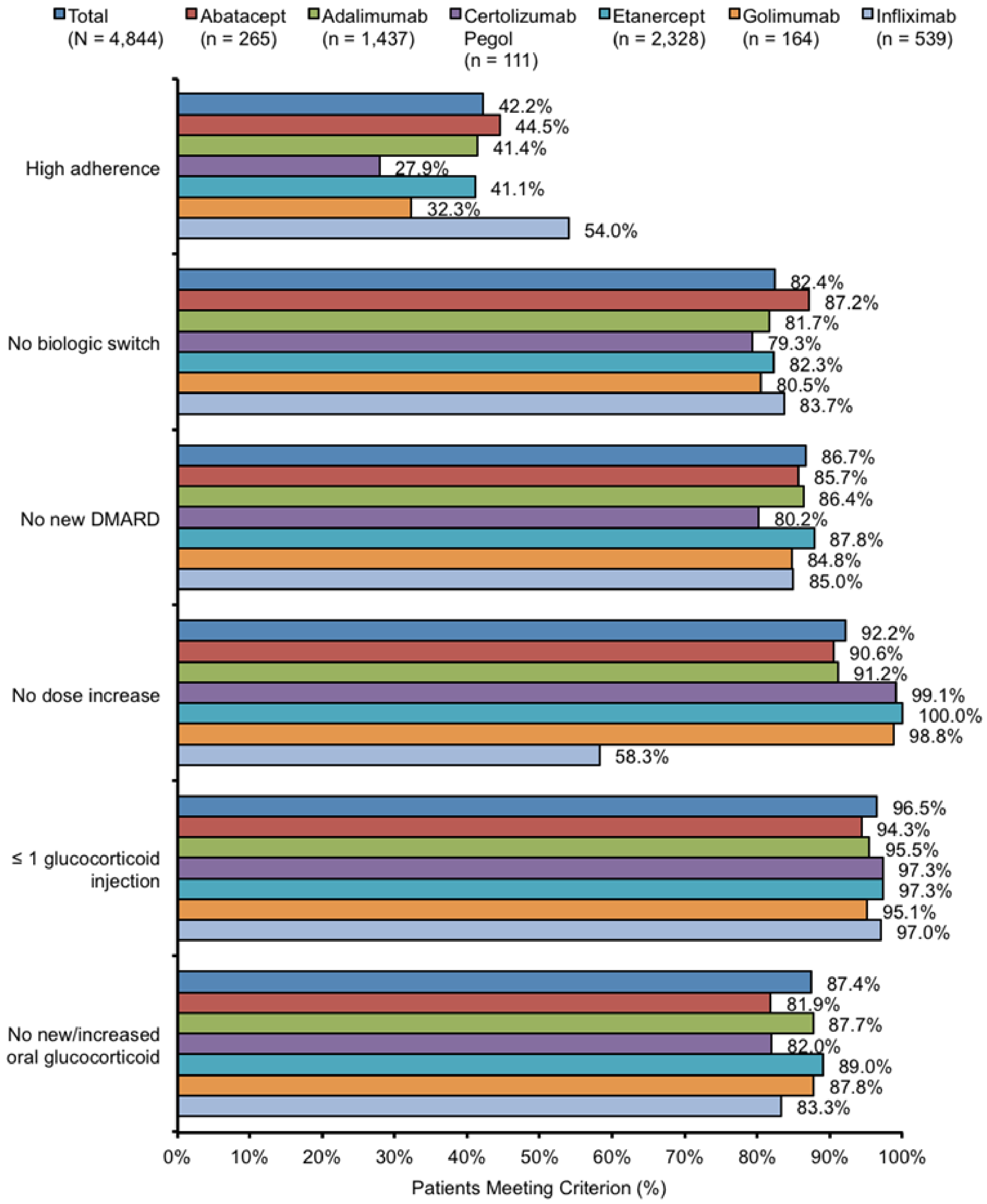

Patients were more likely to fail the criteria for high adherence and no dose increase than the other algorithm criteria Statistical comparisons were not conducted at the individual criteria level

DMARD: disease-modifying antirheumatic drug

**Table 4.** Matrix of Pearson Correlation Coefficients Between Criteria of the Effectiveness Algorithm

| | Pearson Correlation Coefficients (Prob > \|r\| under H0: Rho=0) | | | | | |
|---|---|---|---|---|---|---|
| | **High adherence** | **No biologic switch** | **No new DMARD** | **No dose increase** | **≤ 1 glucocorticoid injection** | **No new/ increased oral glucocorticoid** |
| **High adherence** | 1.0000<br>- | | | | | |
| **No biologic switch** | 0.3260<br>(p<0.001) | 1.0000<br>- | | | | |
| **No new DMARD** | 0.0840<br>(p<0.001) | 0.0836<br>(p<0.001) | 1.0000<br>- | | | |
| **No dose increase** | -0.0981<br>(p<0.001) | 0.0086<br>(p=0.550) | 0.0600<br>(p<0.001) | 1.0000<br>- | | |
| **≤ 1 glucocorticoid injection** | 0.0259<br>(p=0.072) | 0.0362<br>(p=0.012) | 0.0513<br>(p<0.001) | 0.0284<br>(p=0.048) | 1.0000<br>- | |
| **No new/increased oral glucocorticoid** | 0.0914<br>(p<0.001) | 0.1104<br>(p<0.001) | 0.0967<br>(p<0.001) | 0.0470<br>(p=0.001) | 0.0330<br>(p=0.022) | 1.0000<br>- |

DMARD: disease-modifying antirheumatic drug

## DISCUSSION

The average cost per effectively treated patient was significantly lower for etanercept than for other first-line biologics in this analysis using a validated claims-based algorithm in patients with RA from a large managed care population. During the 1-year post-index period, the most commonly failed criterion for all index biologics was the requirement for high adherence. Fewer than half of the patients satisfied this criterion for all index biologics except infliximab (54%), which required only seven physician-administered doses in the first year. Conversely, patients who received infliximab satisfied the criterion for no dose increase at a lower rate (58%) than those who received the other index biologics (90-100%). For each of the other algorithm criteria, patients on each of the index biologics had satisfactory achievement rates of approximately 80% or more.

Similar results were reported in previous studies that used the claims-based algorithm to compute the cost per effectively treated RA patient.[11-15] This study expanded on those findings using newer data (index dates through January 2013) compared with the previous studies (index dates through 2010 or 2011) and different databases.[11-15] Thus, this study represents current practice and includes greater potential exposure to the more recently approved biologics. The previous studies were specific to other payers[11,12] or encompassed a wide range of payers.[13-15] The HIRD represents administrative claims information from the largest health benefits organization in the United States, including lines of business such as health maintenance organizations, point of service plans, preferred provider organizations, consumer-directed health plans, and indemnity plans. Thus, the results can be generalized to approximately 69% of the United States population that was covered by commercial insurance at the time covered in the analysis.[16] Collectively, the results of this study demonstrate the external validity of the results from previous studies,[11-15] over time and across populations.

This study is subject to some limitations. The groups were not adjusted for comparison based on baseline characteristics to mitigate potential bias in the selection of patients for one index biologic versus another. Demographics generally appeared to be similar across the treatment groups, but differences in clinical characteristics such as comorbid conditions and disease severity were not analyzed and could have influenced the effectiveness of biologics. The study was designed to estimate the biologic costs associated with real-world use of biologics in the treatment of patients with RA. In the real-world setting, patient-specific characteristics often influence the selection of a specific biologic; thus, the lack of adjustment for baseline differences enhances the generalizability of the results. Groups that are under-represented in the HIRD are the uninsured and patients who receive government medical coverage, including Medicaid, Medicare, Veterans Administration, and TRICARE. Thus, study results may not be generalizable to these patient populations. Additional studies are warranted to examine the comparative cost-effectiveness of first-line biologics in these populations. Another potential limitation of this analysis was the use of claims data to evaluate biologic effectiveness. Claims data are primarily used for administrative purposes in obtaining reimbursement for services provided to health plan members. As a result, there could be diagnostic and procedural coding inaccuracies causing the misclassification of certain diagnoses, events, or procedures of interest. To mitigate this limitation, the study used a claims-based algorithm that was developed and validated against actual clinical measurements of RA in the United States Veterans Administration population[8] and among commercially-insured patients.[9]

## CONCLUSIONS

Using a claims-based algorithm and data from the largest health benefits organization in the United States, this study demonstrated that the cost per effectively treated patient is lower for etanercept than for other first-line biologics in RA. The findings of this study enhance the generalizability of previous studies across patient populations, where etanercept has been consistently shown to have the lowest cost per effectively treated patient with RA across the biologics evaluated in each individual population. The use of more recent data in this study ensures greater relevance of the findings to current clinical practice.

## ACKNOWLEDGEMENTS

Jonathan Latham of PharmaScribe, LLC (on behalf of Amgen Inc.) and Julie Wang of Amgen Inc. provided medical writing and editing assistance in the preparation of the manuscript.

### Conflict of Interest Declaration

This study was funded by Amgen Inc. N Shah, DH Tang, and DJ Harrison are employees of Amgen Inc., which markets etanercept. T Gu and G Deshpande are employees of HealthCore, which received funding from Amgen Inc. to conduct this study. At the time of the study, DF Eisenberg was an employee of HealthCore.
